# Liver resection *versus* transplantation for multiple hepatocellular carcinoma: a propensity score analysis

**DOI:** 10.18632/oncotarget.20623

**Published:** 2017-09-02

**Authors:** Chuan Li, Jia-Ye Liu, Wei Peng, Tian-Fu Wen, Lu-Nan Yan, Jia-Yin Yang, Bo Li, Wen-Tao Wang, Ming-Qing Xu

**Affiliations:** ^1^ Department of Liver Surgery & Liver Transplantation, West China Hospital of Sichuan University, Chengdu, China

**Keywords:** liver transplantation, liver resection, hepatocellular carcinoma, outcomes, recurrence

## Abstract

The aim of this study was to compare the outcomes of patients with multiple hepatocellular carcinoma (HCCs) after liver resection (LR) versus liver transplantation (LT). Patients who had multiple HCCs without macrovascular invasion and who underwent LT or LR between 2007 and 2013 were reviewed. A propensity score matching model was used to adjust baseline differences between the two groups. A total of 204 patients were selected for the current study, including 137 LR patients and 67 LT patients. During follow-up, 100 patients experienced recurrence, and 78 patients died. The 5-year recurrence-free survival rate was 71.1% for the LT group and 31.1% for the LR group (P<0.001). The 5-year overall survival rate was 73.4% for the LT group and 39.8% for the LR group (P<0.001). Moreover, the LT group had better recurrence-free survival and overall survival rates than the LR group regardless of whether the patients met or exceeded the Milan criteria. The multivariate analysis showed that microvascular invasion and LR were independent risk factors for postoperative recurrence, whereas only LR was associated with an increased incidence of mortality. After applying one-to-one propensity score matching, similar results were observed in the propensity score matching model. Our study suggested that LT provided a better prognosis for patients with multiple HCCs than LR regardless of whether the patients met the Milan criteria.

## INTRODUCTION

Hepatocellular carcinoma (HCC) is the sixth most common malignancy and the third leading cause of cancer-related death worldwide.[1] HCC is mainly associated with chronic hepatitis B virus (HBV) or hepatitis C virus (HCV) infection and accounts for approximately 6% of new cancer patients worldwide.[2, 3] A seroepidemiological survey performed in 2006 showed that the hepatitis B surface antigen carrier rate was 7.18% in the overall Chinese population.[4] Due to this high prevalence, more than half of the HCC cases worldwide occur in China.[5] Many investigations have suggested that multiple HCCs contribute to a poorer prognosis than a single tumor.[6, 7] Liver resection and transplantation are two curative treatments for HCC. According to the Barcelona Clinic Liver Cancer (BCLC) staging system, both liver transplantation and resection are recommended for patients with multiple HCCs within the Milan criteria (i.e., a single tumor up to 5 cm, up to 3 tumors with each tumor no larger than 3 cm, and a lack of vascular invasion or extrahepatic metastasis).[8, 9] Although transcatheter arterial chemoembolization (TACE) is the standard treatment for patients with multiple HCCs outside of the Milan criteria, a number of studies have confirmed that these patients may also benefit from liver resection.[9–11] A randomized comparative trial performed by Lin et al [12] confirmed that overall survival following liver resection was superior to overall survival following TACE for patients with multiple HCCs outside of the Milan criteria. Moreover, some transplant selection criteria allow some patients with multiple HCCs beyond the Milan criteria (e.g., the up-to-seven criteria and the Hangzhou criteria) to undergo LT.[13, 14] However, whether LT or LR offer better outcomes for patients with multiple HCCs is unclear.

The aim of this study is to clarify whether LT or LR is a better curative management practice for patients with multiple HCCs based on their recurrence-free and overall survival.

## PATIENTS AND METHODS

### Study group

Patients with multiple HCCs without macrovascular invasion who underwent LT or LR at our center between 2007 and 2013 were reviewed. Typically, both LR and LT are introduced to patients and/or their close relatives. The choice of treatment was dependent on many factors, including the patient's liver function, portal hypertension, the remnant liver volume, and especially the patient's preference. Currently, our medical insurance does not cover liver transplantation. Patients fulfilling the following criteria were excluded: underwent re-resection; positive surgical margin; presence of other tumor types; received dual graft liver transplantation; and underwent ABO-incompatible liver transplantation. The patients were divided into LR and LT groups based on the treatment received. This study was approved by the ethics committee of West China Hospital.

### Surgical procedure

For the patients who received LR, the liver was exposed via a right subcostal incision with an extension to the upper midline after general anesthesia. Intraoperative ultrasound was routinely used. Hemihepatic vascular occlusion or the Pringle maneuver was utilized to reduce intraoperative bleeding. A CUSA Excel™ device was used for liver transection. Drainage was routinely placed before closure. The donors were ABO blood type compatible and had negative laboratory findings. The donors for living donor liver transplantation (LDLT) were close relatives. Volumetric computed tomography with contrast was administered to evaluate the right hepatic lobes of all donors. The right hepatic lobes of donors without a middle hepatic vein were at least 0.8% of the recipient's weight, and the remaining liver remnant in the donor was at least 40% of the recipient's weight. For patients undergoing LT, the “Mercedes-Benz” incision was used. The liver grafts were preserved and flushed using the University of Wisconsin solution. A venous-venous bypass was not used in all liver transplantations.

### Follow-up

After surgery, the patients were regularly followed-up every three months and monitored using blood cell tests, liver function tests, serum alpha-fetoprotein (AFP) levels, visceral ultrasonography, either computed tomography or magnetic resonance imaging, and chest radiography. Postoperative recurrence was defined as either positive imaging findings compared with the preoperative examinations with or without newly rising tumor marker (AFP) values or confirmation by biopsy or resection.[14] The patients of the two groups were all followed up regularly until death or the termination of this study (September 2016).

### Immunosuppression and antiviral protocols

Immunosuppressive maintenance comprised either tacrolimus or cyclosporine, mycophenolate mofetil, and a steroid after LT. Steroid pulse therapy was conducted in patients with rejection. Whenever possible, the steroid was tailed off as early as possible. For patients receiving LR, anti-viral treatment (entecavir or lamivudine) was administered to patients with a positive preoperative HBV DNA load. For patients who underwent LT, hepatitis B immune globulin was administered to the HBV patients before, during, and after transplantation. Lamivudine was also administered to the hepatitis B surface antigen-positive patients after LT.

### Statistical analysis

All statistical analyses were performed using SPSS 22.0 for Windows. All continuous variables were presented as the mean ± SD and compared using one-way analysis of variance. Categorical variables were compared using either the χ^2^ test or Fisher's exact test. The independent risk factors for recurrence-free survival (RFS) and overall survival (OS) were identified by Cox regression. The Kaplan-Meier method was used to compare the postoperative RFS and OS for the different groups. The differences in the RFS and OS curves were compared using a log-rank test. A P value less than 0.05 was considered significant.

To minimize the risk of selection bias, propensity score matching was used to balance the treatment choice-related characteristics of the two groups. Then, the model was used to provide a one-to-one nearest-neighbor match between patients undergoing LR and LT.

## RESULTS

### Baseline characteristics of the patient cohort

A total of 216 patients were enrolled in the current study. Twelve patients were excluded due to loss of follow-up (9 patients in the LR group and 3 patients in the LT group). Data from the remaining 204 patients were analyzed in this study. A total of 137 patients underwent LR and 67 patients received LT. Among the 67 LT patients, 16 patients received a living donor liver transplant and 51 patients underwent deceased donor liver transplantation. This study included 184 male patients and 20 female patients with a mean age of 49.70±11.19 years. The mean total tumor size was 6.6 ± 1.6 cm, and 112 patients had multiple HCCs outside of the Milan criteria. A total of 73 patients had high preoperative AFP levels (defined as a preoperative AFP level greater than 400 ng/mL).[14] HBV DNA was detected in 78 patients. Microvascular invasion (MVI) was observed in 64 patients, and 31 patients had more than 3 HCCs.

**Figure 1:**
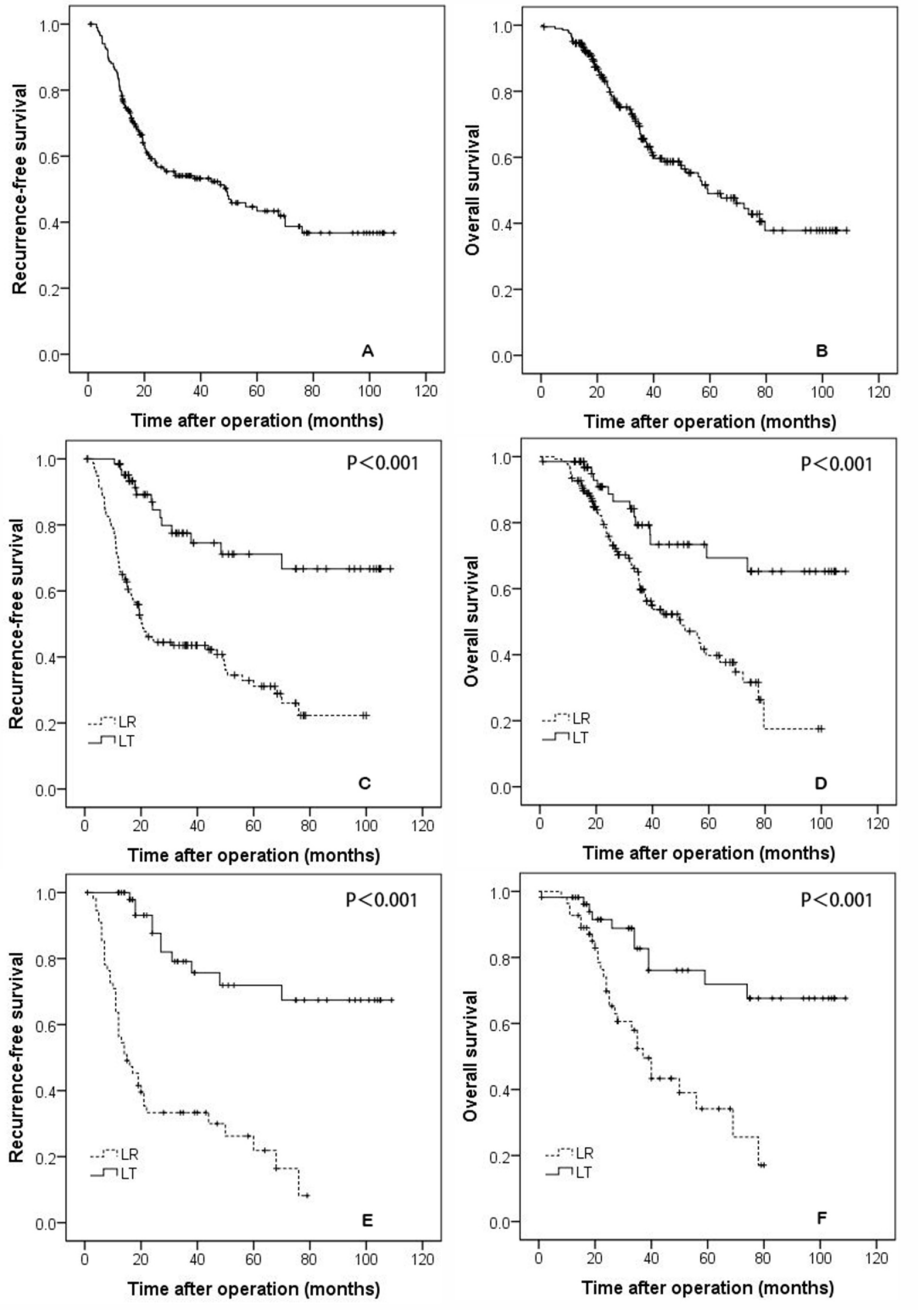
The recurrence-free **A.,** and overall survival **B.,** curves for all patients. The recurrence-free **C.,** and overall survival **D.,** rates for patients who underwent liver resection and transplantation. The recurrence-free **E.,** and overall survival **F.,** rates for propensity-matched patients who underwent liver resection and transplantation.

The mean follow-up time was 38.16±25.19 months (median: 32.42 months). During the follow-up period, 100 patients experienced recurrence, and 78 patients died. The 1-, 3-, and 5-year RFS rates for the whole study cohort were 78.2%, 54.1%, and 43.4%, respectively. The 1-, 3-, and 5-year OS rates for all patients were 94.6%, 65.6%, and 49.0%, respectively (Figure 1A and 1B).

We compared the baseline characteristics of the two groups and found that the LT group had more patients with >3 tumors and more patients with Child-Pugh B and C statuses. There were no significant differences with respect to age, gender, MVI, and tumor size.

### Comparison of RFS and OS for all patients

The 1-, 3-, and 5-year RFS rates were 98.5%, 77.5%, and 71.1% in the LT group and 68.6%, 43.5%, and 31.1% in the LR group, respectively (Figure 1C, *P <* 0.001). The 1-, 3-, and 5-year OS rates of the LT group were 98.5%, 79.3%, and 73.4%, respectively, and were significantly better than the rates of the LR group (92.7%, 59.7%, and 39.8%, respectively, *P* < 0.001; Figure 1D).

### Comparison of the RFS and OS for patients selected for the propensity model

The baseline data for patients selected for the propensity model are shown in Table 1 delineated by group. The baseline characteristics of the two groups were similar in tumor size, tumor number, age, gender, MVI, and differentiation. Patients in the propensity model group undergoing LT had significantly better RFS and OS rates than the patients who received LR. The 1-, 3-, and 5-year RFS rates were 97.9%, 79.1%, and 71.9% for the LT group and 70.9%, 41.5%, and 21.9% for the LR group, respectively (Figure 1E, *P* < 0.001). The 1-, 3-, and 5-year OS rates were 98.2%, 82.7%, and 71.8% for the LT group and 92.7%, 52.4%, and 34.1% for the LR group, respectively (Figure 1F, *P* < 0.001).

**Table 1:** Clinicopathological characteristics of HCC patients before and after one-to-one propensity matching

Variable	All patients			Propensity-matched patients		
	LR (*n*= 137)	LT (*n* = 67)	*n*	LR (*n* = 55)	LT (*n*= 55)	*P*
Age (≥60/<60 years)	27/110	7/60	0.096	8/47	6/49	0.567
Female/male	14/123	6/61	0.776	5/40	6/49	0.751
Total tumor size >5 cm	92/45	42/25	0.528	38/17	35/20	0.545
No. of tumors >3	15/122	16/51	0.016	7/48	7/48	1.000
Milan criteria (yes/no)	61/76	31/36	0.814	24/31	24/31	1.000
Differentiation (poor/well and moderate)	31/106	16/51	0.842	13/42	12/43	0.820
MVI (positive/negative)	44/93	20/47	0.743	15/40	16/39	0.832
AFP (>400/≤400 ng/mL)	52/85	21/46	0.355	20/35	18/37	0.688
HBV DNA load (positive/negative)	57/80	21/46	0.157	20/35	16/39	0.416
BCLC stage A/B	61/76	31/36	0.814	24/31	24/31	1.000
ECOG status (0/1)	132/5	61/6	0.115	52/3	51/4	0.696
Child-Pugh status (A/B/C)	137/0/0	59/7/1	0.001	55/0/0	53/2/0	0.154

### Subgroup analysis based on the Milan criteria

**Figure 2:**
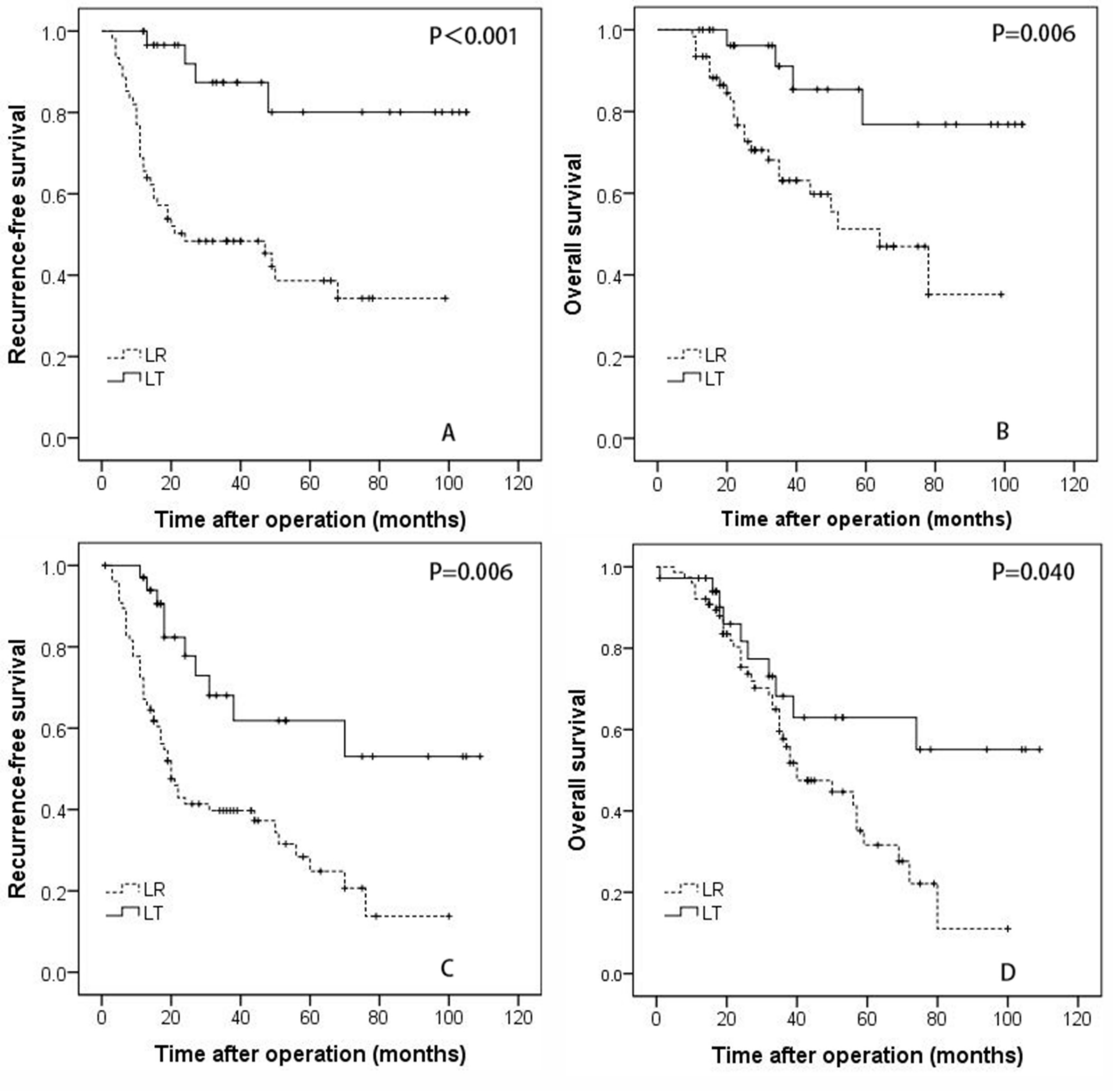
The recurrence-free **A.,** and overall survival **B.** rates for patients with hepatocellular carcinoma within the Milan criteria who underwent liver resection and transplantation. The recurrence-free **C.** and overall survival **D.** rates for patients with hepatocellular carcinoma beyond the Milan criteria who underwent liver resection and transplantation.

In this study, 92 patients with multiple HCCs fulfilled the Milan criteria, including 31 patients who underwent LT and 61 patients who received LR. For the patients with HCCs meeting the Milan criteria, the 1-, 3-, and 5-year RFS rates of the LT group were 100%, 87.4%, and 80.1%, respectively, which were significantly better than the rates of the LR group (68.9%, 48.4%, and 38.6%, respectively, *P* < 0.001; Figure 1A). The 1-, 3-, and 5-year OS survival rates of the LT group (100%, 91.1%, and 85.4%, respectively) patients who were within the Milan criteria were also better than the rates of the LR group patients (93.4%, 63.1%, and 51.2%, respectively, *P* = 0.006; Figure 2B).

In this study, 36 patients in the LT group and 76 patients in the LR group had multiple HCCs beyond the Milan criteria. For patients exceeding the Milan criteria, the 1-, 3-, and 5-year RFS rates of the LT group were 97.1%, 68.0%, and 61.9%, respectively, which were significantly better than the rates of the LR group (72.4%, 39.7%, and 24.8%, respectively; *P* = 0.006; Figure 2C). The 1-, 3-, and 5-year OS survival rates were 97.2%, 68.2%, and 63.0%, respectively, in the LT group and 92.1%, 57.7%, and 31.6%, respectively, in the LR group; the observed differences were significant (*P* = 0.040; Figure 2D).

### Risk factor analysis for postoperative recurrence and survival

As shown in Table 2, the univariate analysis suggested that the presence of MVI, a positive preoperative HBV DNA status, and LR were associated with high postoperative recurrence. However, the multivariate analysis for RFS in all patients suggested that LR (HR = 0.244, 95% CI = 0.138-0.430) and MVI (HR = 1.599, 95% CI = 1.069-2.392) were independent risk factors for postoperative recurrence.

**Table 2:** Univariate and multivariate analysis for RFS in patients with multiple HCCs undergoing LR or LT

Variable	Number	Univariate analysis			Multivariate analysis		
		HR	95% CI	*P*	HR	95% CI	*P*
Age (≥ 60/<60 years)	34/170	1.048	0.502-2.189	0.900			
Female/male	20/184	0.957	0.380-2.410	0.926			
No. of tumors (>3/≤3)	31/173	1.401	0.647-3.035	0.392			
Total tumor size (>5/≤5 cm)	134/70	1.028	0.576-1.832	0.926			
Milan criteria (yes/no)	92/112	1.625	0.932-2.835	0.086			
MVI (yes/no)	64/140	2.020	1.105-3.693	0.021	1.599	1.069-2.392	0.022
AFP (>400/≤400 ng/mL)	73/131	0.935	0.527-1.658	0.819			
HBV-DNA status (positive/negative)	78/126	1.917	1.081-3.399	0.025			0.127
Differentiation (poor/well and moderate)	47/157	0.996	0.519-1.911	0.990			
LR/LT	67/137	0.157	0.079-0.310	<0.001	0.244	0.138-0.430	<0.001

As presented in Table 3, the univariate analysis indicated that LR, a positive preoperative HBV DNA status, and failure to meet the Milan criteria were potential risk factors related to poor OS. However, in the Cox proportional hazards model, only LR (HR = 0.364, 95% CI = 0.202-0.655) was associated with an increased mortality rate.

**Table 3:** Univariate and multivariate analyses for OS in patients with multiple HCCs undergoing LR or LT

Variable	Number	Univariate analysis			Multivariate analysis		
		HR	95% CI	*P*	HR	95% CI	*P*
Age (≥ 60/<60 years)	34/170	1.000	0.469-2.133	1.000			
Female/male	20/184	0.586	0.232-1.480	0.254			
No. of tumors (>3/≤3)	31/173	1.622	0.705-3.731	0.252			
Total tumor size (>5/≤5 cm)	134/70	1.178	0.647-2.144	0.592			
Milan criteria (yes/no)	92/112	1.843	1.032-3.291	0.038			0.054
MVI (yes/no)	64/140	1.400	0.766-2.558	0.273			
AFP (>400/≤400 ng/mL)	73/131	0.698	0.383-1.272	0.240			
HBV DNA status (positive/negative)	78/126	1.871	1.048-3.342	0.033			0.174
Differentiation (poor/well and moderate)	47/157	1.127	0.579-2.194	0.725			
LR/LT	67/137	0.301	0.153-0.593	<0.001	0.364	0.202-0.655	0.001

## DISCUSSION

HCC ranks as the third most frequent cancer-related death worldwide due to its aggressive nature.[1] Many studies have confirmed that multiple tumors are associated with an increased incidence of postoperative recurrence and a decreased survival rate.[6, 7] However, the optimal management strategy for patients with multiple HCCs is not well established. Our study suggested that liver transplantation provided better RFS and OS for patients with multiple HCCs without macrovascular invasion.

Multiple HCCs may be either intrahepatic metastasis from a primary HCC or multicentric in origin.[15, 16] Nagasue et al [17] suggested that multiple HCCs might all develop intrahepatic recurrence within 5 years after LR due to their multicentric origin. Many studies reported that the most common site for recurrence was the remaining liver.[16, 17] Some studies suggested that early recurrence (within the first 2 years after the operation) after liver resection might be metastasis arising from the primary HCC, whereas late recurrence (more than 2 years after operation) might have a multicentric origin.[18, 19] Wu and colleagues suggested that multiple HCCs were an independent risk factor for both early and late recurrence after liver resection. LT removes the entire affected liver and can eradicate micro-metastasis of the remaining liver. Moreover, LT not only removes the tumors but also cures any background liver diseases. In contrast, LR only resects the tumor and has no effect on background liver diseases.

Some investigations suggest that LR may offer long-term survival rates that are similar to the LT survival rates for patients with HCC within the Milan criteria.[20, 21] However, these studies included either solitary tumors up to 5 cm or no more than 3 tumors with each tumor no more than 3 cm.[20, 21] In this study, we only included multiple tumors. We confirmed that the outcomes of patients with multiple HCCs who underwent LT were better than the outcomes of patients who underwent LR. Fan et al [22] confirmed that patients with a single tumor up to 5 cm had better 5-year survival rates than patients with polynodular tumors (2-3 nodules, each ≤3 cm). Poon et a l[23] also suggested that having multiple tumors was an independent risk factor associated with an increased recurrence rate and mortality for patients with HCC within the Milan criteria after liver resection or transplantation.

TACE is the standard treatment for patients with multiple HCCs outside of the Milan criteria.[9] However, increasing evidence has shown that liver resection may provide a better prognosis for patients with multiple HCCs outside of the Milan criteria.[24, 25] A randomized comparative trial suggested that liver resection had better OS for patients with multiple HCCs outside of the Milan criteria than TACE.[12] A multicenter study also confirmed that patients with resectable HCC could benefit from LR over loco-regional therapy regardless of the cancer stage.[26] Recently, a systematic review and meta-analysis performed by Liu et al. confirmed that LR offered improved OS compared to TACE for patients with multiple HCCs beyond the Milan criteria.[27] A large systematic review and meta-analysis performed by Qi et al [28] also confirmed that LR provided better OS than TACE for patients with HCC. In Qi et al's subgroup meta-analysis, LR offered better OS for patients with HCC beyond BCLC stage A.[28] Moreover, some investigations suggested that LT could achieve outcomes for well-selected patients with HCC beyond the Milan criteria that were similar to the outcomes achieved with patients with HCC within the Milan criteria.[29] Our study suggested that LT offered better RFS and OS than liver resection for patients with multiple HCCs outside of the Milan criteria.

Our study suggested that MVI was associated with a high incidence of postoperative recurrence but not with OS. Previous studies confirmed that MVI was a strong prognostic factor for postoperative recurrence after either LR or LT.[22, 30] However, Shah et al [31] suggested that macrovascular invasion but not MVI was related to poor long-term survival after LT. The study performed by Vivarelli et al [32] also indicated that there was no relationship between postoperative OS and MVI for patients with HCC after LT. Moreover, Chan et al [33] reported that LT doubled the chances of a cured status for patients with HCC, with MVI, and within up-to-7 criteria compared with LR.

Although several published studies have compared the outcomes of LR versus LT for HCC,[23, 34–37] many of these studies focused on patients with early HCC.[23, 37] These studies also only included patients with single HCC.[23, 37] In contrast to these studies, our study only involved patients with multiple HCCs.

In conclusion, our study suggested that patients with multiple HCCs had better RFS and OS following liver transplantation compared with liver resection regardless of whether they met or exceeded the Milan criteria.

## Abbreviations

(AFP): alpha-fetoprotein
(BCLC): Barcelona Clinic Liver Cancer
(HBV): hepatitis B virus
(HCV): hepatitis C virus
(HCC): hepatocellular carcinoma
(LR): liver resection
(LT): liver transplantation
(PSM model): propensity score matching model
(TACE): transcatheter arterial chemoembolization
